# Up-regulated expression of *CD147* gene in malignant bone tumor and the possible induction mechanism during osteoclast formation

**DOI:** 10.1590/1414-431X20186948

**Published:** 2018-07-23

**Authors:** Suchi Qiao, Chang Liu, Weijie Xu, WuBuLi AZhaTi, Cheng Li, Zhiwei Wang

**Affiliations:** 1 Department of Orthopedics, Changhai Hospital, The Second Military Medical University, Shanghai, China; 2 Changzheng Hospital, The Second Military Medical University, Shanghai, China; 3 Department of Orthopedics, Fuzhou General Hospital, Fuzhou, China

**Keywords:** Bone cancer, RT-PCR, Western blot, RANKL, Human

## Abstract

It is increasingly evident that the microenvironment of bone can influence cancer phenotype in many ways that favor growth in bone. CD147, a transmembrane protein of the immunoglobulin (Ig) superfamily, was identified independently in different species and has many designations across different species. However, expression levels of CD147 mRNA in bone cancer have not been described. In this study, we have used real-time fluorescence quantification (RT-PCR) to demonstrate CD147 expression in malignant bone cancer and benign bone tumor tissues. The results suggested that the expression of *CD147* gene was significantly up-regulated in malignant bone cancer. Moreover, we found that over-expressed RANKL progressively enhanced osteoclast formation up to 48 h, which suggested that RANKL could promote the formation of osteoclast, indicating that both CD147 and RANKL play important roles in the formation of osteoclasts. Furthermore, the expressions of four osteoclast specific expression genes, including *TRACP*, *MMP-2*, *MMP-9* and *c-Src*, were analyzed using RT-PCR. The results indicated that four osteoclast-specific expression genes were detectable in all osteoclast with different treatments. However, the highest expression level of these four osteoclast-specific expression genes appears in the CD147+ RANKL group and the lowest expression level of these four osteoclast-specific expression genes appears with si-RANKL treatment. Characterization of the role of *CD147* in the development of tumors should lead to a better understanding of the changes occurring at the molecular level during the development and progression of primary human bone cancer.

## Introduction

Bone is a very dynamic tissue resulting from coordinated phases of formation and resorption called bone remodeling. In addition to its role in phosphocalcic homoeostasis, the bone remodeling process is necessary for bone growth and renewal of cellular and extracellular matrix components to adapt bone organization to the various biological and mechanical constraints (1–3). Multiple myeloma also has a strong preference for growth and metastasis in the bone marrow. The most common sites of bone metastases are the spine, ribs, pelvis, proximal femur, and skull. Breast cancer preferentially metastasizes to the lungs and bones, whereas prostate cancer almost exclusively metastasizes to the bones (4).

Bone metastases can be classified as osteolytic with significant bone destruction, osteoplastic due to excess bone formation, or a mixed phenotype can occur (5). Metastasis to the bones is facilitated by the fenestrated structure of the bone marrow sinusoid capillaries, high blood flow in the areas of red marrow, and adhesive molecules on tumor cells that bind to the bone marrow stromal cells such as osteoblasts and osteoclasts as well as the bone matrix (6). These cell types play an important role in bone remodeling and niche structure (7). The bone marrow microenvironment includes osteoblastic (endosteal) and vascular niches, which provide an environment that supports hematopoietic and non-hematopoietic stem cells (8).

Bone homeostasis is maintained by balanced production of osteoblasts and osteoclasts. Disruption of this balance can convert normal niches into metastatic niches. Disseminated tumor cells in the bone marrow can persist in a dormant state for many years until they progress to a macrometastatic lesion (9). The clinical challenges associated with bone metastasis are deleterious effects such as bone pain, fractures, life-threatening hypercalcemia, spinal cord compression, and other nerve compression syndromes (10). Deeper understanding of the process of bone metastasis and identification of targets for prevention and treatment is, therefore, an objective of paramount importance.

Osteoblasts and osteoclasts are crucial for interactions with tumor cells in the context of their colonization of the bone marrow. Osteoclasts are multinucleated cells that arise from precursor cells of the monocyte-macrophage lineage. They de-mineralize bone by dissolving calcium phosphate crystals and producing acid and are involved in bone resorption through degradation of the newly-exposed extracellular matrix (ECM) by secreted proteases. Osteoclasts adhere to bone surface and the spectrum of factors involved in their activation may depend on tumor type. Osteoblasts are specialized, terminally differentiated mononuclear cells differentiated from mesenchymal stem cells and are involved in bone reconstruction by generation of new ECM and deposition of calcium phosphate crystals into the interstices of the matrix (11). Prerequisite for the generation of osteolytic metastases is the activation of osteoclasts. To this end, osteoblasts secrete receptor activator of NF-κB ligand (RANKL), which interacts with osteoclast precursors displaying RANK receptor on their surface, resulting in their activation and finally maturation into functional osteoclasts.

Osteoblasts also produce osteoprotegerin (OPG), a soluble decoy receptor which can block RANK/RANKL signaling by scavenging of RANKL. Thus, the activation of osteoclasts is triggered by the balance between RANKL and OPG (12–13). RANKL also can induce factors involved in migration, invasion, and angiogenesis such as matrix metalloproteinases 1 and 9 (MMP1, MMP9), matrix metalloproteinase inducer EMMPRIN/CD147, intercellular adhesion molecule-1, interleukin 6 and 8 (IL6, IL8), and vascular endothelial growth factor (14) and decrease the expression of metastasis suppressor serpin 5b/maspin (15). RANKL can also promote the function of regulatory T-cells and macrophages (16).

Osteolysis is based on a self-perpetuating signaling system (vicious cycle) that is maintained by mitogenic factors for tumor cells such as transforming growth factor-β, insulin-like growth factor-1, fibroblast growth factors, platelet derived growth factors, and Ca-ions released from demineralized bone as well as parathyroid hormone-related peptide (PTHrP) derived from tumor cells. PTHrP acts as a promotor of osteolysis by osteoclasts (17).

CD147, a transmembrane protein of the immunoglobulin (Ig) superfamily, was identified independently in different species and has many designations across different species such as M6, neurothelin, 5A11, HT7, OX-47, CE9, EMMPRIN, basigin, and gp42 (18–20). Soluble CD147 also has been detected in microvesicles (exosomes) (21–22). Homotypic interactions may play an important role regarding the mode of action of MMP induction since it has been shown that recombinant CD147 fusion proteins can interact homotypically (23). However, interaction of CD147 with an as yet unidentified receptor might also be responsible for the phenomena as described above.

CD147 can be induced in tumor cells by epidermal growth factor receptor mediated signaling (autocrine mechanism) (24). Controlled degradation of the extracellular matrix is a prerequisite for tumor invasion and metastasis. Transfection of breast cancer cells with CD147 resulted in increased tumor growth and metastasis after implantation into the mammary gland and correlated with high levels of MMP-2 and MMP-9 (25). The molecular mechanisms of these phenomena have been poorly resolved. Glycosylated CD147 was shown to be a prerequisite for MMP induction at the transcriptional level (25). Induction of MMPs by CD147 was prevented with a monoclonal antibody directed against CD147.

In all bone cancers, a strong relationship between tumor cells and bone micro-environment has been clearly established, facilitating the tumor development and/or the metastatic process. These specific communication pathways have strongly stimulated research and development programs to design new drugs to treat oncologic bone diseases and have led specifically to the development of therapies targeting RANKL. The current study represents a comprehensive evaluation of CD147 expression pattern in bone cancer. The results of this study will provide the necessary background information for further research on the regulation and function of this intriguing peptide.

## Material and Methods

### Clinical samples

Malignant bone tumor and benign bone tumor tissue samples from 64 patients (mean age of 57.12±11.31 years, male:female 39:25) were collected at the time of dissection from patients at the Changhai Hospital from July 2014 to December 2016. The biopsies were frozen and stored at –80°C until preparation of total RNA. All tumor tissues were histologically confirmed to be from bone tumor. The study was approved by the ethics committee of Changhai Hospital, China.

### Cell treatment

RAW264.7 cells were purchased from ATCC (USA) and maintained in RPMI 1640 with 10% (v/v) FBS (Invitrogen, USA). RAW264.7 cells in 6-well plates (2×10^7^ per well) in a humidified incubator with 5% CO_2_ at 37°C were supplemented with RANKL (PeproTech, 100 ng/mL) and then transfected with pcDNA 3.1/CD147 or si-RANKL. All cells were divided into five groups with different treatments: Control: no treatment; CD147+RANKL: over-expression of CD147 and RANKL (100 ng/mL); RANKL: RANKL (100 ng/mL); CD147+si-RANKL: over-expression of CD147 and knock-down of RANKL; si-RANKL: knock-down of RANKL alone; and constructing CD147: the full length of CD147 was amplified and cloned into pcDNA3.1 vectors (Invitrogen). si-RANKL was used for further studies, which were synthesized by GenePharma (China). The sequence of si-RANKL is listed in Table 1. Lipofectamine 2000 (Invitrogen) was used to transfect oligonucleotides and constructs into cell lines according to the manufacturer's instructions. The infection efficiency was confirmed by qRT-PCR or western blot 48 h after transfection.

**Table 1.** Primer sequences used in the study.

Gene	Primers
*si-RANKL*	R: 5-GTGCAGAAATGGCGAGAATAC-3 F: 5-TGCACCACCAACTGCTTAGC-3
*GAPDH*	R: 5-GGCATGGACTGTGGTCATGAG-3 F: 5-GGCCCTGTCACTCCTGAGAT-3
*MMP-2*	R: 5-GGCATCCAGGTTATCGGGGA-3 F: 5-TGGACGATGCCTGCAACGTG-3
*MMP-9*	R: 5-GTCGTGCGTGTCCAAAGGCA-3 F: 5-GGCTGTGAAGTCGTCAGAACAC-3
*CD147*	R: 5-ACCTGCTCTCGGAGCCGTTCA-3 F: 5-CGTCTCTGCACAGATTGCAT-3
*TRACP*	R: 5-CGTCTCTGCACAGATTGCAT-3 F: 5-CAT CCA AGCCTC AGACCCA-3
*c-Src*	R: 5-TGACACCACGGCATACAGC-3 F: 5-GCACCACACCTTCTACAATG-3

### TRACP staining

Tartrate-resistant acid phosphatase (TRACP) staining was performed by a modification of a previously described method (26) and the amount of TRACP positive multinucleated cells was calculated. Cells were fixed and stained for TRACP according to manufacturer instructions after 7 days of culture in the presence of RANKL or CD147.

### Western blot analysis

Total cellular protein in RAW264.7 cells with different treatments was isolated by the addition of 1% PMSF and RIPA lysis buffer (50 mM Tris-HCl (pH 7.4) (Sigma-Aldrich, USA), 150m M NaCl, 1% NP-40, 0.1% SDS). After boiling with SDS-PAGE sample buffer for 5 min, the samples were prepared for sodium dodecyl sulfate-polyacrylamide gel electrophoresis. Then, proteins were transferred onto a polyvinylidene difluoride membrane (Millipore, USA). After being blocked for 1 h at room temperature, the membrane was incubated with a 1:1000 dilution of rabbit polyclonal anti-mouse TRAF6, NFATc1, and β-actin antibody (ABGENT, USA) overnight. Before detected with an ECL chemiluminescence detection kit (Advansta, USA), proteins were incubated with the corresponding secondary antibody for 1 h at room temperature. The bands were obtained by GeneGnome 5 (Synoptics Ltd., UK).

### RT-PCR

Total RNA was extracted from tissues using Trizol (Invitrogen). Reverse transcription of mRNA from malignant bone tumor, benign bone tumor, and RAW264.7 cells was carried out in 100 µL final volume from 400 ng total-RNA using high capacity cDNA Archive kit (Applied Biosystems, USA) according to manufacturer's instructions. The levels of mRNAs were measured by real-time quantitative RT-PCR (qRT-PCR) using Bio-Rad IQ5 system, and primers were utilized as described in Table 1. Reactions were performed in 50 µL volumes containing SYBR Green PCR master mix (Perkin-Elmer Biosystems, USA). Real-time PCR was performed using Bio-Rad IQ5 system in 96-well optical plates. Thermal cycling conditions were as follows: 2 min at 50°C and 10 min at 95°C, followed by 40 cycles at 95°C for 30 s, 60°C for 30 s, and 72°C for 2 min. Data were collected using the ABI analytical thermal cycler (Applied Biosystems). RNA expression was calculated based on a relative standard curve with ΔΔct method.

### Statistical analysis

The measurement data are reported as means±SD. Independent continuous variables were compared using the *t*-test. P<0.05 was considered to be statistically significant. All calculations were made using SPSS 18.0 (SPSS Inc., USA).

## Results

### CD147 was up-regulated in malignant bone tumor

In order to investigate the conditions of CD147 expression in malignant bone tumor, CD147 cDNA was selected for further verification of differential expression by RT-PCR. Using specific primers designed for the CD147 cDNA clone (Table 1), RT-PCR was performed on malignant bone tumor and benign bone tumor tissues.

**Figure 1.**
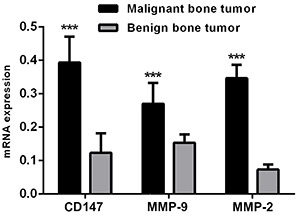
CD147, matrix metalloproteinases (MMP)-9, and MMP-2 mRNA relative expression *vs* GAPDH in malignant and benign bone tumor. Data are reported as means±SD. ***P<0.001, compared with the benign bone tumor (*t*-test).

The result of this RT-PCR analysis is shown in Figure 1. CD147 mRNA was detected in malignant bone tumor and benign bone tumor tissues. CD147 expression levels were markedly up-regulated over half of the expression levels in the benign bone tumor tissues as well as the MMP-2 and MMP-9 expression levels. On the contrary, CD147 expression levels in the benign bone tumor tissues samples analyzed were lower than that in malignant bone tumor. Collectively, our results indicated that abnormal up-regulation of CD147 was confirmed in human malignant bone tumor.

### Over-expression of CD147 can effectively promote osteoclast formation

**Figure 2.**
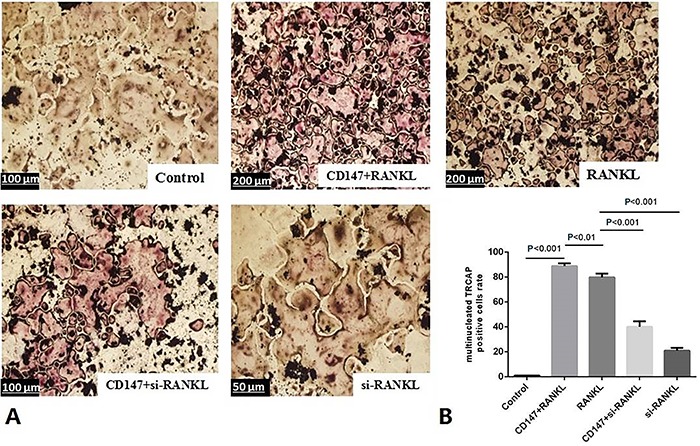
*A*, Tartrate-resistant acid phosphatase (TRACP)-positive osteoclast formation in mice that received different treatments. TRACP. *B*, Multinucleated TRACP positive cells per group, compared with the *t*-test. Data are reported as means±SD. RANKL: receptor activator of NF-κB ligand.

Transfection was validated by RT-PCR that assessed increased ΔCt levels in transfected cells compared with Control and Blank (data not shown). We found that over-expressed RANKL progressively increased osteoclast formation up to 48 h of transfection (Figure 2), which suggested that RANKL can promote the formation of osteoclast. Up to 48 h, CD147+RANKL group had significantly increased processes of osteoclast formation compared with the other groups (P<0.05).

Knock-down of RANKL can effectively reduce the osteoclast formation compared with over-expression of RANKL. Therefore, it can be summarized that over-expression of CD147 and RANKL can promote the osteoclast formation.

### Western blot of key factors in RANKL signaling pathway with over-expression of CD147

**Figure 3.**
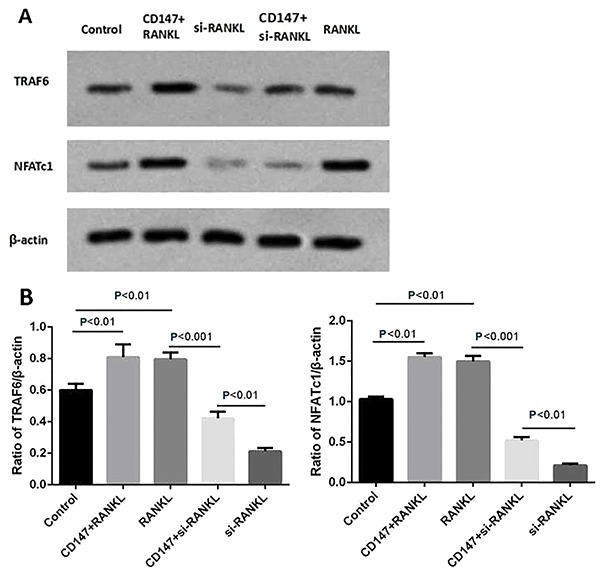
Western blot analysis of the effects of *CD147* gene expression on the key factors in the RANKL signaling pathway. *A*, Western blot analyses of TNF receptor-associated factor-6 (TRAF6) and nuclear factor of activated T-cells cytoplasmic 1 (NFATc1e). *B*, Ratio of TRAF6 and NFATc1 to β-actin. Data are reported as means±SD and compared with the *t*-test. RANKL: receptor activator of NF-κB ligand.

We evaluated the expressions of key factors such as TNF receptor-associated factor-6 (TRAF6) and nuclear factor of activated T-cells cytoplasmic 1 (NFATc1) using western blot (Figure 3) (27). In the Control group (48 h), there was no significant difference in the protein expression of the two key factors. The expressions of TRAF6 in si-RANKL and CD147+si-RANKL group were down-regulated compared with control groups. Meanwhile, the expressions of NFATc1 in si-RANKL and CD147+si-RANKL groups were also down-regulated compared with control groups. However, the expression of NFATc1 in CD147+ RANKL group was significantly up-regulated compared with Control group. The results mentioned above suggested that over-expressed *CD147* and *RANKL* genes can retain the expression of key factors in RANKL signaling pathway (*in vitro*).

### RT-PCR analysis of osteoclast-specific expression genes with different treatments

The expressions of four osteoclast-specific expression genes, including *TRACP*, *MMP-2*, *MMP-9*, and *c-Src*, were analyzed by using RT-PCR, in which GAPDH was employed as a control for both cDNA quality and efficiency of the PCR amplification.

**Figure 4.**
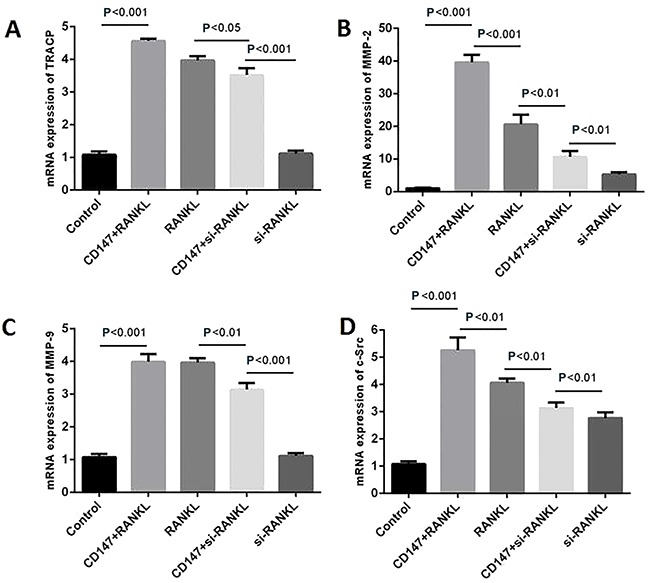
RT-PCR analysis of tartrate-resistant acid phosphatase (TRACP), matrix metalloproteinases (MMP)-2, MMP-9, and c-Src with over-expressed *CD147* gene in osteoclasts. GAPDH was used as reference gene. Data are reported as means±SD and compared with the *t*-test. RANKL: receptor activator of NF-kB ligand.

As shown in Figure 4, four osteoclast-specific expression genes were detectable in all osteoclasts with different treatments. However, the highest expression level of these genes appeared in the CD147+ RANKL group and the lowest appeared in the si-RANKL treatment.

## Discussion

CD147 is a member of the immunoglobulin family of receptors. Members of this family play a role in intercellular communication involved in many immune-related functions, differentiation, and development. CD147 plays a role in spermatogenesis, lymphocyte activation, and expression of monocarboxylate transporters, and has been identified as a regulatory subunit of the γ-secretase complex in Alzheimer's disease amyloid β-peptide production (28–30). Some of these insights were obtained from the study of cd147-/- mice. These animals are defective in MMP regulation, spermatogenesis, lymphocyte responsiveness, and neurological functions at the early stages of development. Such female mice are infertile due to failure of implantation and fertilization (31). CD147 is involved in the transport of the MCT-1 and MCT-3 to the plasma membrane since reduced accumulation of these transporters has been observed in the retina of cd147 knockout mice.

A functional role of CD147 in cell adhesion is supported by its involvement in the blood-brain barrier and its interactions with integrins. CD147 has been implicated in many pathological processes, such as rheumatoid arthritis, experimental lung injury, atherosclerosis, chronic liver disease induced by hepatitis C virus, ischemic myocardial injury, and heart failure (32). Treatment of transplant patients with a CD147 antibody was effective due to inhibition of T-cell activation (33). From this study, RT-PCR analysis showed that CD147 mRNA was detected in malignant bone tumor and benign bone tumor tissues. CD147 expression levels were markedly up-regulated over half of the expression levels in the benign bone tumor tissues.

A high incidence of CD147 expression in different cancer entities through tissue microarrays and monoclonal antibodies (mAb) MEM-M6/1 and HIM6 was noted in a systematic investigation (34). Several of the 2348 and 608 tissue samples covering 129 tumor types and 76 normal tissues, respectively, were investigated for their CD147 status with these antibodies. CD147 expression was found in 112 out of 129 tumor entities with the following incidences: squamous cell carcinomas (60–100%), pancreatic cancer (87%), chromophobic kidney cancer (83%), hepatocellular carcinoma (83%), medullary breast cancer (83%), and glioblastoma multiforme (79%).

A homogeneous expression of CD147 was found in tumors such as squamous cell carcinoma of different organs and mesotheliomas. The following normal tissues scored positively for CD147 expression: proliferatively active and differentiating epithelial cells, myocardial cells in the left heart ventricle, and vascular endothelial cells of the brain. Interestingly, CD147 isoforms differing in presence or absence of Lewis X glycan structures were found on breast cancer cells. Another investigation of expression and function of CD147 as a cancer-associated biomarker made use of mAb HAb18 (IgG1) (35). Several of the 28 tissue microarrays and 1117 pathological sections of breast tissue samples were analyzed. The incidence of CD147 expression was: cancer of the liver 80% (n=20), lung 62% (n=90), stomach 66% (n=44), colon 58% (n=19), rectum 59% (n=17), breast 64% (n=1055), brain 90% (n=52), esophagus 87% (n=16), ovary 75% (n=40), urinary bladder 85% (n=41), skin (squamous cell carcinoma) 58% (n=41), larynx 85% (n=63), and kidney 73% (n=33), and 30% of sarcomas such as osteo, chondro- and fibrosarcoma (n=102). Staining was ranked as weak, moderate, and strong. Strong staining was observed in 20% of breast, ovarian, and brain tumors. The level of CD147 expression was correlated with survival of the patients in a retrospective study of 106 patients with infiltrating ductal carcinoma of the breast (35). Up-regulation of CD147 also has been noted in glioma, laryngeal squamous cell, ovarian, renal cell, and skin carcinoma (36–37). CD147 was described as a marker of poor diagnosis in serous ovarian and bladder carcinomas (38). Collectively, CD147, the pleiotropic glycoprotein, plays a role in every stage of the progression of malignant tumors, including invasion, metastasis, angiogenesis, survival, and multidrug resistance. However, the prognostic role of CD147 in ovarian cancer is still conflicting. Given the multiple roles of CD147 in ovarian cancer development, a potential therapeutic strategy targeting CD147 to prevent ovarian cancer progression would be useful. In fact, CD147 is considered a biomarker of many tumors and is currently the subject of clinical trials in hepatocellular carcinomas (39). The use of CD147 as a new diagnostic and prognostic marker for bone cancer needs further study.
